# Islet transplantation outcomes in type 1 diabetes and transplantation of HLA-DQ8/DR4: results of a single-centre retrospective cohort in Canada

**DOI:** 10.1016/j.eclinm.2023.102333

**Published:** 2023-12-13

**Authors:** Shareen Forbes, Anne Halpin, Anna Lam, Don Grynoch, Richard Parker, Luis Hidalgo, David Bigam, Blaire Anderson, Khaled Dajani, Tatsuya Kin, Doug O'Gorman, Peter A. Senior, Patricia Campbell, A.M. James Shapiro

**Affiliations:** a Clinical Islet Transplant Programme, University of Alberta, Edmonton, Canada; b Department of Surgery, University of Alberta, Edmonton, Canada; c Queen's Medical Research Institute, BHF Centre for Cardiovascular Science, University of Edinburgh, Scotland, UK; d Alberta Precision Laboratories, University of Alberta, Laboratory Medicine and Pathology, University of Alberta, Edmonton, Canada; e Division of Endocrinology and Metabolism, Department of Medicine, University of Alberta, Edmonton, Canada; f Alberta Diabetes Institute, University of Alberta, Edmonton, Canada; g Edinburgh Clinical Trials Unit, Usher Institute, University of Edinburgh, Scotland, UK; h Department of Surgery, University of Wisconsin, Madison, WI, USA

**Keywords:** Type 1 diabetes, Islet transplantation, HLA antigens, HLA-DQ8, HLA-DQ2-DQA1∗05

## Abstract

**Background:**

In solid organ transplantation, HLA matching between donor and recipient is associated with superior outcomes. In islet transplantation, an intervention for Type 1 diabetes, HLA matching between donor and recipient is not performed as part of allocation. Susceptibility to Type 1 diabetes is associated with the presence of certain HLA types. This study was conducted to determine the impact of these susceptibility antigens on islet allograft survival.

**Methods:**

This is a single-centre retrospective cohort study. This cohort of transplant recipients (n = 268) received islets from 661 donor pancreases between March 11th, 1999 and August 29th, 2018 at the University of Alberta Hospital (Edmonton, AB, Canada). The frequency of the Type 1 diabetes susceptibility HLA antigens (HLA-A24, -B39, -DQ8, -DQ2 and–DQ2-DQA1∗05) in recipients and donors were determined. Recipient and donor HLA antigens were examined in relation to time to first C-peptide negative status/graft failure or last observation point. Taking into account multiple transplants per patient, we fitted a Gaussian frailty survival analysis model with baseline hazard function stratified by transplant number, adjusted for cumulative islet dose and other confounders.

**Findings:**

Across all transplants recipients of donors positive for HLA-DQ8 had significantly better graft survival (adjusted HRs 0.33 95% CI 0.17–0.66; p = 0.002). At first transplant only, donors positive for HLA-DQ2-DQA1∗05 had inferior graft survival (adjusted HR 1.96 95% CI 1.10–3.46); p = 0.02), although this was not significant in the frailty analysis taking multiple transplants into account (adjusted HR 1.46 95% CI 0.77–2.78; p = 0.25). Other HLA antigens were not associated with graft survival after adjustment for confounders.

**Interpretation:**

Our findings suggest islet transplantation from HLA-DQ8 donors is associated with superior graft outcomes. A donor positive for HLA-DQ2-DQA1∗05 at first transplant was associated with inferior graft survival but not when taking into account multiple transplants per recipient. The relevance of HLA-antigens on organ allocation needs further evaluation and inclusion in islet transplant registries and additional observational and interventional studies to evaluate the role of HLA-DQ8 in islet graft survival are required.

**Funding:**

None.

**Research in context**

**Evidence before this study**

Current guidelines recommend islet transplantation to treat intractable problematic hypoglycaemia. Although an efficacious procedure, there are no detailed analyses relating how specific Type 1 diabetes susceptibility HLA antigens in donors and between donors and recipients influence islet graft survival outcomes. We searched MEDLINE, Embase, and Scopus for research articles reporting long-term outcomes following allogeneic pancreatic islet transplantation published between Jan 1, 2000, and May 1, 2023, using the search terms “pancreatic islet transplant” AND “islet transplantation” AND “islet transplant” AND “donor Type 1 diabetes susceptibility antigens”. Publications detailing HLA-antigens that are not considered to be susceptibility HLA-antigens for development of Type 1 diabetes were not included. Abstract presentations were excluded. No publications were identified on this specific topic.

**Added value of this study**

To our knowledge, this study from a single centre following islet transplantation is the largest report of Type 1 diabetes susceptibility HLA antigens in donors and between donors and recipients that has studied their impact on islet graft survival outcomes. This study of allogeneic islet transplant recipients, incorporates comprehensive testing of several Type 1 diabetes susceptibility HLA antigens including HLA-A24, -B39, -DQ8, -DQ2 and–DQ2-DQA1∗05 and relates these antigens to long term follow-up outcomes of islet graft survival (C-peptide >0.1 nmol/L throughout follow-up). The report takes into account and adjusts for multiple donor and recipient factors including induction and immunosuppressive agents known to impact islet transplantation outcomes including T cell depleting agents, anti-TNF-α agents (etanercept) and anti- IL-1Ra (anakinra) therapy and factors in time from transplantation. It examines other potential confounders including recipient GAD autoantibody status and their relationship with donor HLA-antigens received. The study identified that receiving islets from donors positive for HLA-DQ8 (-DR4) has a highly significant protective effect on islet graft survival. In adjusted analyses, there was also some evidence that receiving HLA- DQ2-DQA1∗05 (-DR3) at first transplant only, may lead to poorer graft survival but there was no impact overall of receiving this antigen across multiple transplants.

**Implications of all the available evidence**

Evidence suggests that islet transplantation is an efficacious therapy for selected patients with Type 1 diabetes with proven safety and sustained metabolic control. There is no evidence to select or avoid specific HLA-antigens for transplantation. The donor HLA-DQ8 findings support further studies to examine the mechanism by which islet graft survival is improved following islet transplantation. Such further research may then lead to the development and adoption of new immunotherapies into clinical trials of beta cell preservation and replacement to extend graft longevity potentially. It may translate into administering specific peptides based on HLA-DQ8 to patients undergoing islet transplantation including stem cell-derived islets which would need evaluation in clinical trials. Our findings may impact on further research into gene editing stem cell-derived islets for future transplantation. The relevance of HLA-antigens on organ allocation needs further evaluation and inclusion in islet transplant registries.

## Introduction

Islet transplantation is a clinically proven intervention for a subset of patients with Type 1 diabetes (T1D) debilitated with recurrent life-threatening hypoglycaemia. The procedure can substantially improve glycemic control and temporarily eliminate the need for exogenous daily insulin injections.^1^ Stabilisation of blood glucose control, prevention of severe hypoglycaemia, restoration of symptoms of hypoglycaemia and reduced progression of diabetes-related microvascular complications^2^ positively impacts quality-of-life.^3^, ^4^, ^5^ However, the procedure comes at a cost of the need for chronic immunosuppression, and the treatment is not available universally. Major factors limit the application of islet transplantation including scarcity of appropriate organ donors.

Due to islet losses secondary to ischaemic injury and the islet purification process,^6^ islet transplant recipients typically require islet infusions from ≥two donor pancreases to achieve the goal of receiving ≥10,000 islet equivalent units (IEQ)/kg. This number of islets increases the likelihood of achieving insulin independence^7^ but the prevalence of sustained insulin independence is low and graft survival is variable.^8^, ^9^, ^10^

It is not understood why some transplant recipients remain insulin independent for over 20 years after intraportal islet transplantation, whereas many require exogenous insulin following islet transplantation after a few months to years. As well as the risk of rejection of allotransplants, loss of insulin-producing beta cells mediated by autoimmunity may be an important contributing factor.

HLA antigens are protein molecules expressed on cell surfaces which mediate immune responses. There are numerous loci of interest including class I antigens (A, B and C) and class II antigens (DR, DQ and DP). HLA-A, -B and DR are traditionally typed in solid organ transplantation with matching of the donor and recipient at HLA-A, B and DR loci. Such matching is associated with better graft function, improved graft survival and fewer rejection episodes in donor transplantation of kidney, heart, lung and bone-marrow.^11^^,^^12^ In islet transplantation HLA matching is not part of routine pancreas organ allocation algorithms, and due to the paucity of data, there is little evidence regarding HLA matching between donor and recipient.

In T1D, loss of beta cells occurs secondary to the targeting of islet cell autoantigens by autoreactive T cells.^13^ Predisposition for T1D derives from HLA class II alleles specifically HLA-DRB1∗04 (DR4), DQA1∗03 DQB1∗03:02 (DQ8), HLA-DRB1∗03:01 (DR17) and DQA1∗05:01-DQB1∗02:01 (DQ2).^14^ HLA class I antigens may also be associated with an increased risk for T1D including HLA-A24,^14^ HLA-B39^14^ (see www.hla.alleles.org) but at lower prevalence. It is not known how these HLA antigens impact islet transplant outcomes.

The aim of this retrospective study was to determine if recipient and, or, donor T1D susceptibility HLA antigens were associated with long-term islet graft dysfunction.

## Methods

### Study design and participants

**Fig. 1:**
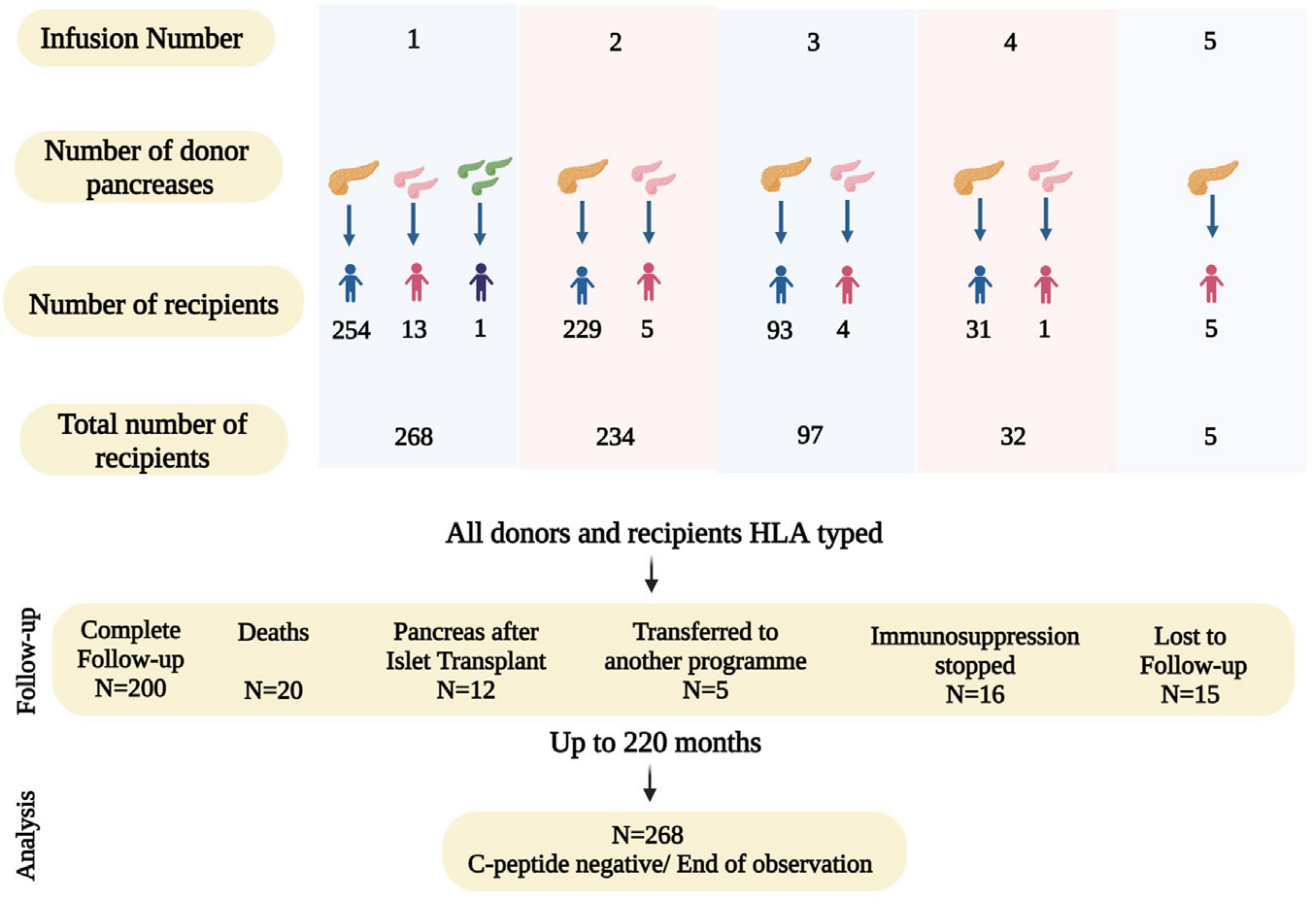
A total of 636 transplant infusions were performed from 661 donor pancreases in 268 recipients. A range of one to five transplants were performed. In transplant one, of the 268 recipients, 254 recipients received islets from one donor, 13 recipients received islets from two donors and one recipient received islets from three donors. In transplant two, of the 234 recipients, 229 received islets from one donor, five received islets from two donors. In transplant three, of the 97 recipients, 93 received islets from one donor, four received islets from two donors. In transplant four, of the 32 recipients, 31 received islets from one donor, 1 received islets from two donors. In transplant five, all five recipients received islets from single donors. Donor and recipients were HLA typed. Follow up was for up to 220 months and loss to follow-up and other reasons for data censoring are shown. Immunosuppression stopped (cancer N = five, side effects/patient choice N = four, failed graft N = seven). Time of i) next transplant or ii) C-peptide negative status or iii) end of observation period was analysed as applicable. Figure created with Biorender.com.

This was a single centre retrospective cohort study of people with T1D undergoing allogeneic islet transplantation at the University of Alberta Hospital (Edmonton, AB, Canada) between 11th March 1999 to 29th August 2018. Participants were >18 years old with T1D > five years and an undetectable stimulated C-peptide (<0.1 nmol/L). The main indication for islet transplantation was T1D complicated by recurrent severe hypoglycaemia despite optimised conventional management.^15^ Patients were invited to take part in this study approved by the institutional health research ethics board (PRO000001120 and PRO00087040) and written informed consent was obtained. Baseline data including age, sex and duration of diabetes, HbA1c pre transplant, insulin dose and anthropometric data was recorded. Fig. 1 illustrates donor pancreas numbers and recipient islet infusions over the study period.

### Allocation of organs for transplant and release criteria

Pancreases donated after brain death (DBD) and after circulatory death (DCD) procured nationally across Canada were offered to ABO compatible recipients. The process of islet isolation is as previously reported.^15^

Where insulin independence was attained after a single islet transplant a clinical decision was made to not relist for a further infusion. Otherwise, recipients were relisted after metabolic assessment at two-four weeks following their first islet transplant. Islet product release criteria are listed (Supplemental Appendix). Number of islets infused, time between transplants, induction agents and immunosuppression was recorded.

### Perioperative and post-transplant patient management

Islets were transplanted by percutaneous delivery into the liver intraportal circulation under radiological guidance and managed as previously described.^15^ Induction therapy with T cell depleting (TCD) agents was commenced following confirmation of islet release criteria; alemtuzumab was commenced <2 h before and thymoglobulin 24–48 h prior to the recipients’ first islet transplantation. In subsequent transplants, induction therapies were repeated unless the lymphocyte count was <0.1 × 10^9^ cells/L, in which case basiliximab was given.

### Clinical, metabolic and HLA antigen assessments and outcome measures

Clinical visits were undertaken at one, three, six and 12 months after first islet transplant and yearly thereafter and the below tests performed.

### Metabolic tests and analyses

A standardised 90-min mixed-meal tolerance test with Ensure HP was performed^15^ and glucose and C-peptide analyzed.^7^^,^^10^ Graft failure was defined as a stimulated C-peptide <0.1 nmol/L. If graft failure was clinically suspected, levels were measured locally and the results recorded centrally.

### HLA typing analyses and antibody screening

#### HLA typing

Low to medium resolution HLA class I and II typing was performed using the One Lambda Thermo Fisher Micro SSP™ or the LabType™ assay. Manufacturer's protocols for amplification and electrophoresis were followed. HLA A, B, C, DRB1, DRB3, 4, 5, DQA1, DQB1 and DPA1 and DPB1 were typed.^16^ For all recipients and donors HLA-A24 and HLA-B39, HLA-DQ2, HLA-DQ8, and HLA-DQB1∗02-DQA1∗05 (HLA-DQ2∗-A05), were analysed. HLA-DQ2∗A05 typing was started a little later than for the other antigens, consequently, there are less data points for this antigen.

#### Antibody screening

HLA antibody screening protocols evolved with time but comprised a screening assay and at least one single antigen bead assay prior to transplant^17^ (see Supplemental Appendix).

#### Autoantibody screening

Anti-glutamic acid decarboxylase IgG (GAD) antibodies were determined using an ELISA and were performed pre-transplant in recipients after December 2011 and available in 114 of the 268 recipients.

The primary outcome measure was the time to first C-peptide negative status, or last observation point in relation to recipient and donor HLA antigens.

### Statistical analysis and data handling

The baseline demographic data on the recipients was >95% complete. HLA recipient and donor antigen data was >93% complete. A maximum of n = 268 was available for analysis.

HLA handling: The recipient and donor were considered either positive or negative for a specific antigen. Where islets had been received from multiple donors in a specific infusion all donor antigens were considered. For the survival analysis, when considering all transplants, the cumulative numbers of antigens were classified as 1 where there was ≥1 positive (or 0 otherwise) and when considering matching of donor with recipient antigens across multiple transplants these were classified as 1 where there was ≥1 match (or 0 otherwise).

All available data was entered and data was inferred where possible based on results in other categories: if the donor or recipient was known to be negative for a specific HLA-antigen but the corresponding recipient or donor HLA antigen status was not known then it was assumed that there was no match between donor and recipient. If across multiple transplants there was incomplete donor antigen data, but if ≥1 antigen was positive this was noted in the model; if however there were no positive antigens and data was missing then this was left as missing data as the status could not be inferred with certainty.

The frequency of HLA antigens at high risk for the development of T1D: HLA-A24, HLA-B39, HLA-DQ2, HLA-DQ8, HLA-DQ2∗-A05 were calculated: where one or two HLA antigens were present, a positive status was assigned and compared between donors and recipients by Fisher's Exact Test. The calculated Panel Reactive Antibody (cPRA) is an estimate of the percentage of donors to whom a recipient has HLA antibodies or unacceptable antigens.^18^ The cPRA calculator used was from the Canadian Transplant Registry and is further described (Supplemental Appendix).^18^

Islet graft survival and effect of donor antigens and donor and recipient antigen matches: C-peptide positive status was termed graft survival and conversely C-peptide negative status, graft failure. Donor HLA antigens at first transplant and also cumulative donor HLA antigens across all transplants were examined in relation to the time to first C-peptide negative status or last observation point. Recipient and donor HLA antigens were examined in relation to matching of specific antigens at first transplant and then at subsequent transplants in relation to the time to first C-peptide negative status or last observation point.

Log-rank testing was used to test differences between survival curves according to HLA-antigen donor and recipient/donor matching status with no adjustments for covariates. Unadjusted HRs and 95% confidence intervals were reported for all HLA antigens.

A stratified Cox Gaussian frailty survival analysis, adjusted for cumulative islet numbers, recipient age and sex and induction with T cell depleting agents (TCDs) ± TNF-α inhibitors and immunosuppression including with MTOR inhibitors, Calcineurin inhibitors (CNI), anakinra an IL-1 receptor antagonist and mycophenolate mofetil (MMF; a reversible inhibitor of inosine monophosphate dehydrogenase (IMPDH)) were performed. Baseline hazard functions were stratified by the transplant number, and a Gaussian frailty term was included in the models. These agents were selected as they are frequently used in islet transplantation. Models were also run without anakinra and MMF but including all other covariates.

By convention and in order to take into consideration the effect of each transplant, the time between first transplant and each subsequent transplant was considered from the time of first transplant as the reference point.

In sensitivity analysis, an extreme value imputation method was used whereby the same frailty models were fitted as above except that any missing values for HLA antigens were replaced by 0 or 1 depending on whether graft failure occurred as an outcome. Thus, we were able to give a range of all possible values of the hazard ratios and 95% confidence intervals regardless of our underlying assumptions regarding the missing data.

In secondary post-hoc analysis, a standard Cox survival analysis model was fitted to the total graft survival time from first transplant until C-peptide failure or last observation time, examining relevant first transplant donor and donor-recipient HLA antigen matches and adjusting for all confounders.

The results were reported as HRs and 95% confidence intervals.

Relationships with allele homozygosity and era of treatment, where the data was divided into tertiles based on year of transplant (n = 89, n = 62 and n = 117 in eras one-three respectively), were also examined.

Autoantibody and HLA-antigen analyses: GAD autoantibody status in recipients was examined in relation to specific HLA antigens to ensure the sample was representative of the Type 1 diabetes population. GAD autoantibody positive recipients were subsequently examined in relation to “high risk” and “protective” donor HLA-antigens received using Fishers exact test in order to check that the autoantibody positive or negative recipients did not receive by chance transplants with the high-risk or protective HLA antigens respectively.

Donor data and islet characteristics were examined in relation to HLA-antigens and distribution compared by Fishers exact test.

HLA data handling and statistical methods are described in further detail (Supplemental Appendix).

A p-value of <0.05 was regarded as statistically significant. Statistical analyses were performed with STATA version 15.0 (StataCorp, College Station, TX) and R version 4.3.1.^19^ for the Cox survival and missing data sensitivity analyses.

### Role of the funding source

All authors had full access to all the data in the study and accept responsibility for the decision to submit for publication. There was no funding source for this study.

## Results

A total of n = 268 recipients received n = 636 islet infusions from n = 661 donor pancreases between March 11th, 1999 and August 29th, 2018. Prior to 2006, islets from up to three donated pancreases were pooled and transplanted together accounting for numbers of donated pancreases transplanted being greater than the number of infusions.

Table 1 shows islet transplant recipient characteristics in islet transplant alone (ITA) recipients (n = 246) and islets after kidney (IAK) transplant recipients (n = 22). Eighty five per cent of recipients were of white ethnicity and 43% were male and 57% female. HbA1c levels were greater in IAK versus ITA recipients 8.8 (8.3–9.6) versus 8.2 (7.4–9.1) % (p = 0.02).

**Table 1:** Recipient characteristics.

Recipients	All	ITA	IAK
n	268	246	22
M:F (%M)	116:152 (43)	104:142 (42)	12:10 (55)
Age (yrs)	48.8 (41.2–56.0)	48.8 (41.3–56.0)	48.5 (38.9–55.4)
Age >35 yrs (n (%))	242 (90%)	221 (90%)	21 (95%)
Duration of Diabetes (yrs)	30.2 (22.7–40.4)	30.0 (22.3–39.4)	36.8 (26.9–42.8)
Pre transplant weight (Kg)	72.1 (63.8–80.2)	72.1 (64.0–80.2)	72.7 (60.1–82.9)
Pre transplant height (m)	1.68 (1.62–1.76)	1.68 (1.62–1.76)	1.68 (1.64–1.73)
Pre transplant BMI (kg/m2)	24.8 (22.9–27.7)	24.8 (23.0–27.6)	25.3 (21.8–29.4)
HbA1c (pre tx) (%)	8.2 (7.5–9.1)	8.2 (7.4–9.1)	8.8 (8.3–9.6)^a^
Insulin (U/day)	39 (31–49.5)	39 (31–49)	41 (34–52)
Insulin (U/kg)	0.55 (0.46–0.69)	0.54 (0.46–0.69)	0.57 (0.50–0.67)
Pre-transplant DSA	6	6	0

Data is median (IQR) unless otherwise stated.

p values are differences between IAK versus ITA recipients.

IAK, Islets after kidney; ITA, Islet transplant alone.

a p < 0.05, difference between groups in HbA1c.

The majority of pancreases (n = 661) were donated after brain death (96.8%). Donor details are shown (Table 2).

**Table 2:** Donors characteristics.

Transplant	Donor pancreases n	DBD n (%)	CIT hours	Age years	Sex M(%)	BMI Kg/m^2^	HbA1c (%)
1	283	276 (97.5)	8.5 (5.3–11.5)	50 (42–58)	169 (59.7)	28.3 (24.9–32.3)	5.7 (5.4–5.9)
2	239	233 (97.5)	9.3 (5.0–11.5)	49 (40–57)	151 (63.2)	28.0 (24.7–32.4)	5.6 (5.4–5.9)
3	101	97 (96.0)	10.1 (5.1–12)	55 (43–60)	55 (54.4)	27.4 (24.5–31.8)	5.6 (5.4–5.9)
4	33	30 (90.9)	11.3 (5.5–13.3)	52.5 (45–61)	22 (66.6)	28.6 (23.1–31.2)	5.8 (5.6–6.0)
5	5	4 (80.0)	4.3 (4.0–11.8)	41 (40–56)	3 (60.0)	26.4 (25.8–26.6)	5.3 (5.1–5.4)
1–5	661	640 (96.8)	9.0 (5.0–11.8)	50 (42–58)	398 (60.2)	28 (24.7–32.2)	5.6 (5.4–5.9)

Number of donated pancreases for each transplant and overall.

For DBD status and sex, data is absolute numbers (%). All other data is median (IQR).

DBD—donation after brain death, a minority of pancreases were donated after cardiac death.

CIT, cold ischaemic time; BMI, Body Mass Index.

Donor characteristics did not differ by HLA-antigen group (all p > 0.05).

Recipients received between one to five transplant infusions; the majority received two sequential transplants. Follow up was for up to 220 months and complete in 200 participants (Fig. 1).

GAD autoantibody titres were greatest in recipients positive for HLA-DQ2∗-A05 and HLA-DQ8 and lowest in those negative for both antigens (Table S1).

The median islet mass per transplant was 423,933 (359,419–507,425) IEQs and 5998 (5253–6982)IEQ/kg with no difference between ITA versus IAK recipients (Tables S1 and S2A). The cumulative number of islets received over five transplants was <2.8 million IEQs or 40,469 IEQ/kg (Figure S1A and S1B). Islet isolation parameters including culture time, viability and purity are shown (Table S2B); there were no significant differences in donor characteristics or islet isolation parameters according to the different donor HLA-antigens (all p > 0.05).

The time between infusion one and two was significantly shorter: 149 (64–331) days (p = 0.01) versus the time intervals between other transplants (Table S3).

Of note 67.5% received a TCDs ± etanercept and 30.6% anakinra with their first transplant (Table S4). Immunosuppression maintenance (as part of immunosuppression regimen) included: 94% on CNI; 52.6% on an mTOR inhibitor; 41.4% on an mTOR inhibitor with CNI; 39.6% on MMF; 39.6% on MMF and CNI.

### High-risk T1D HLA antigens

The presence of HLA-A24, -B39, -DQ8, -DQ2 and -DQ2∗-A05 antigens was higher in T1D recipients versus organ donors (Table 3; Figure S2), with the HLA antigen prevalence in donors representative of the Canada-wide population (Table 3).

**Table 3:** Prevalence of specified HLA antigens in transplant recipients, study donors and Canada-wide population.

HLA	All recipients N = 268 N (%)	All donors N = 661 N (%)	Donor HLA Frequency-(Canada) (%)
A24	64 (23.9)	129 (19.5)	(19.2)
Missing data (%)	(0)	(0.61)	–
B39	23 (8.6)	30 (4.5)	(4.2)
Missing data (%)	(0)	(0.5)	–
DQ8	150 (56.0)	141 (21.3)	(17.5)
Missing data (%)	(1.1)	(1.5)	–
DQ2∗A05	149 (56.2)	131 (19.8)	–
Missing data (%)	(1.1)	(6.6)	
DQ2	164 (61.9)	243 (36.7)	(38.4)
Missing data (%)	(1.1)	(1.51)	–

Legend: For each HLA antigen, the prevalence of ≥1 antigen n (%) was expressed. The percentage of missing data for each of the antigens is also shown. For the Canada wide population (%) only are displayed. Study recipient and donor frequencies were compared by Fisher's exact test. For the Canada wide donor HLA frequency the cPRA calculator was used: shorturl.at/ewPQ8 based on Canadian Transplant Registry that estimates the percentage of Canadian deceased organ donors with whom a transplant candidate may be incompatible.

– Not able to determine (note—DQ2 alone not a high risk antigen).

All p < 0.005∗∗ for recipients versus donors.

### Donor and recipient HLA antigens and effect on graft survival

**Fig. 2:**
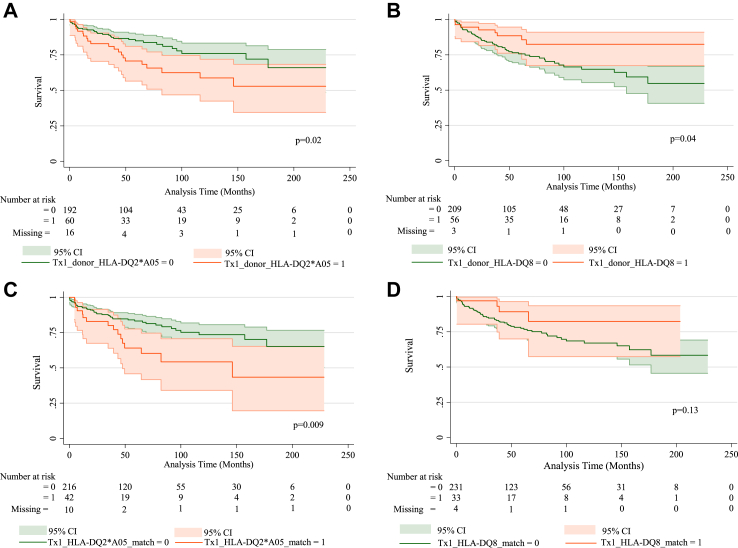

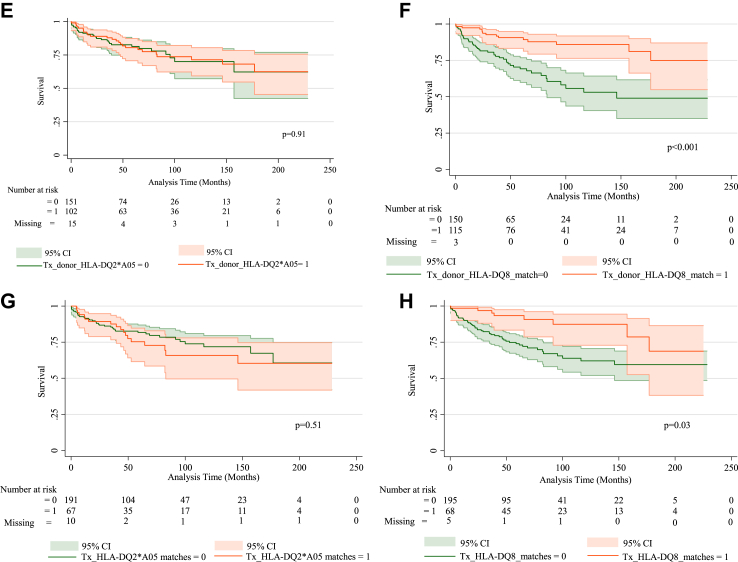
A) 1st transplant and HLA-DQ2∗A05 absent or present in donor: absence confers benefit in graft survival; green—absence of HLA-DQ2∗A05 in donor/s at first transplant, orange—presence of HLA-DQ2∗A05 in donor/s at first transplant. B) 1st transplant and HLA-DQ8 absent or present in donor: presence confers benefit in graft survival; green—absence of HLA-DQ8 in donor/s at first transplant, orange—presence of HLA-DQ8 in donor/s at first transplant. C) HLA-DQ2∗A05 match between donor and recipient absent or present across 1st transplant only: no match confers benefit in graft survival; green—no match in HLA-DQ2∗A05 between donor and recipient at first transplant, orange—match in HLA-DQ2∗A05 between donor and recipient at first transplant. D) HLA-DQ8 match between donor and recipient absent or present across 1st transplant only: no difference observed; green—no match in HLA-DQ8 between donor and recipient at first transplant, orange—match in HLA-DQ8 between donor and recipient at first transplant. E) HLA-DQ2∗A05 absent or present in donors across all transplants: no difference observed; green—absence of HLA-DQ2∗A05 in donors across all transplants, orange—presence of HLA-DQ2∗A05 in donors across all transplants. F) HLA-DQ8 absent or present in donors across all transplants: presence confers benefit in graft survival; green—absence of HLA-DQ8 in donors across all transplants, orange—presence of HLA-DQ8 in donors across all transplants. G) HLA-DQ2∗A05 match between donor and recipient absent or present across all transplants: no difference observed; green—no match in HLA-DQ2∗A05 between donor and recipient across all transplants, orange—match present in HLA-DQ2∗A05 between donor and recipient across all transplants. H) HLA-DQ8 match between donor and recipient absent or present across all transplants: match confers benefit in graft survival; green—no match in HLA-DQ8 between donor and recipient across all transplants, orange—match present in HLA-DQ8 between donor and recipient across all transplants. Proportion ± 95% CI shown. Donor HLA-antigen presence and HLA-antigen match between donor and recipient was recorded when ≥1 antigen and, or, ≥1 match respectively.

The overall graft survival rate is shown (Figure S3A). Recipient HLA typing did not show any relationship with graft survival (Table 4). In contrast, receiving donor positive HLA DQ2∗-A05 at first transplant, was associated with diminished graft survival, unadjusted HR 1.92 (95% CI 1.12–3.33; p = 0.02; Fig. 2A, Table S5) with a sustained negative effect after adjusting for all confounders (Table S6). The presence of HLA DQ2∗-A05 in recipient and donor at first transplant, was associated with diminished C-peptide graft survival: unadjusted HR 2.11 (95% CI 1.19–3.75; p = 0.01; Fig. 2C, Tables S5 and S6) although this relationship was weakened by the adjustment for confounders (Table S6). Over multiple transplants however, HLA DQ2∗-A05 positive donors and donor and recipient matches did not appear to impact graft survival (Table 4).

**Table 4:** Adjusted Hazard Ratios of HLA antigens for time to first c-peptide negative status using a stratified Cox frailty survival analysis of recipient and donor data across all transplants.

Subgroup	+ve antigen/total	HR	95% CI	p
Recipient HLA antigens				
HLA-A24	64/268	0.57	0.28–1.17	0.13
HLA-B39	23/268	0.73	0.24–2.23	0.58
HLA-DQ8	150/265	0.91	0.49–1.68	0.76
HLA-DQ2	164/265	0.74	0.39–1.41	0.36
HLA-DQ2∗A05	149/265	0.99	0.53–1.86	0.98
Donor HLA antigens (≥1 across all donors)^a^				
HLA-A24	105/268	0.69	0.35–1.34	0.27
HLA-B39	29/268	1.92	0.78–4.76	0.16
HLA-DQ8	115/265	0.33	0.17–0.66	**0.002**
HLA-DQ2	166/266	1.51	0.81–2.79	0.19
HLA-DQ2∗A05	102/253	1.46	0.77–2.78	0.25
Recipient and Donor Matched HLA antigens (≥1 match across all donors)^b^				
HLA-A24	32/266	0.41	0.14–1.19	0.10
HLA-B39	4/268	0.88	0.12–6.14	0.86
HLA-DQ8	68/263	0.31	0.13–0.75	**0.009**
HLA-DQ2	110/267	1.19	0.66–2.18	0.56
HLA-DQ2∗A05	67/258	1.72	0.89–3.35	0.11

Legend: Hazard Ratios (95% CI) in recipients were examined to first C-peptide negativity or to end of study period. Recipient HLA antigen status shown.

Donor HLA-antigens received and Recipient and Donor matched HLA antigens are shown in relation to total number of recipients with available data across all transplants. A frailty adjustment was used to correct for multiple transplants from time from first transplant. The model was adjusted for the confounders: islet numbers, participant age and sex, use of T cell depeleting agents ± etanercept, anakinra, MTORI + CNI and MMF.

In the subgroup of all recipients with at least one HLA-DQ8, the HRs for the recipient and donor matched at HLA-DQ8 was 0.96 (95% CI 0.24–3.77, p = 0.95). Therefore, as there is little difference between the risk of C-peptide failure among those with matched DQ8 antigen it suggests that it is the donor antigen that confers the benefit.

p < 0.005 values are denoted in bold.

HR: hazard ratio. MTORI: MTOR inhibitor; CNI: calcineurin inhibitor; MMF: mycophenolate mofetil.

a At least one positive donor antigen across all transplants.

b At least one match across all transplants (specific donor and recipient antigen both positive).

Across all transplants, recipients that received donor islets positive for HLA-DQ8 had superior graft survival (Fig. 2B, D, F and H), including after adjustment for confounders: HR 0.33 (95% CI 0.17–0.66; p = 0.002; Table 4). In the missing imputation analysis these highly statistically significant results were retained for the donor HLA-DQ8 antigen (Table S7). Where recipient and donor were both HLA-DQ8 positive across the transplants there were beneficial effects on C-peptide survival on adjusted analyses (Table 4) which were supported by the results from the missing imputation analysis (Table S7). In the subgroup of all recipients receiving at least one HLA-DQ8 antigen, who by definition were either HLA-DQ8 positive or negative, the adjusted HRs for the recipient and donor matched HLA-DQ8 was 0.96 (95% CI 0.24–3.77, p = 0.95 Table 4), suggesting that it is the donor antigen that confers the benefit.

In further exploratory analyses, that were not adjusted for confounders, recipients who received a 1st transplant from donors that were positive for HLA-DQ2∗-A05 (and negative for -DQ8) vs. those who received transplants from donors positive for both HLA-DQ2∗-A05 and–DQ8 had inferior outcomes of borderline significance (p = 0.057; Figure S3B). There was a protective effect of receiving HLA-DQ8 in the transplant when the donor and recipient at the first transplant were matched for HLA-DQ2∗-A05 (p = 0.04; Figure S3C). Recipients that received HLA-DQ8 showed similar outcomes regardless of whether they did or did not receive HLA-DQ2 across all transplants (Figure S3D).

Homozygosity at the HLA-DQ2∗-A05 typings at first transplant was present in only eight donors but graft survival was significantly shorter than expected (p = 0.02). Homozygosity at the HLA-DQ8 antigen at first transplant was present in three donors and therefore analyses were not performed. Over eras of treatment, where donor at first transplant and recipient were both positive for HLA-DQ2∗-A05, graft survival was shortest in the earliest era (p = 0.03). At this specific time the use of TCDs was significantly lower versus later eras. There was no effect of era on other alleles.

On unadjusted analyses, the presence of HLA-A24 conferred benefit on islet graft survival (Table S5); in the adjusted Cox frailty survival model the presence of donor HLA-A24 was not statistically significant (Table 4), although confidence intervals appeared to exclude clinically important negative effects of this antigen.

There was no effect of HLA-DQ2 on graft survival. HLA-B39 was at low prevalence among donors and no effects on graft function were seen with wide confidence intervals (Table 4).

Of note when anakinra and MMF were taken out of the Cox frailty survival models, statistical significance was unchanged for all the HLA-antigens.

GAD positive autoantibodies were not more prevalent in recipients of HLA-DQ2∗A05 and conversely GAD autoantibody negative status was not associated with recipients of HLA-DQ8 antigens (all p > 0.05).

In subanalyses of the ITA group alone, all results were similarly statistically significant; the IAK group alone were underpowered to perform separate statistical analyses.

The prevalence of pre-transplant donor specific antibodies (DSA) was low (Table 1) and DSA to HLA-DQ8 and–DQ2∗-A05 were not detected.

## Discussion

In this study including the world's largest data series of islet transplant recipients carried out at a single center, we have demonstrated that islet transplantation outcomes although efficacious are highly variable with transplant success strongly associated with numbers of islets transplanted.^7^^,^^15^^,^^20^

Our data demonstrates, as expected, a higher prevalence of HLA-A24 and HLA-B39, HLA-DQ2, HLA-DQ8, HLA-DQ2∗-A05 in people with T1D versus donors with normal glucose tolerance (the latter representative of the Canada-wide donor population).

Recipient susceptibility HLA antigens alone did not impact graft survival. Previous studies have shown that recipient's positive for HLA-A24 have inferior graft outcomes independent of HLA-antigens received and immunosuppression with autoantibody surges post-transplant defining those most at risk; the study was smaller and the group examined younger than in our study which may explain differences in outcome.^21^

Unexpectedly, the presence of donor HLA-DQ8 across transplants, conferred protection of islet graft survival even after adjustment for recipient factors, induction and immunosuppressive agents including TCD agents, calcineurin and mTOR inhibitor immunosuppression which are known to impact on graft survival.^20^

HLA typing methods have evolved from serological methods to distinguish HLA antigens to DNA-based molecular techniques. HLA-DR3 (17) (DRB1∗03:01) is in linkage disequilibrium with DQA1∗05 DQB1∗02 and HLA-DR4 is in linkage disequilibrium with DQ8 in people with T1D. HLA-DR4 may also be associated with DQ7 but this typing combination is not associated with T1D, underlining the importance of the inclusion of HLA-DQ with HLA typing.^22^ In ∼90% of white children diagnosed with T1D, either the HLA-DR4/DQ8 or the HLA-DR3/DQ2 haplotype is found,^23^ consistent with our data which showed a prevalence of 89.5% for either haplotype in our transplant recipients. HLA class II antigens including HLA-DQ2∗-A05 and HLA-DQ8 are expressed by human beta cells in T1D and may be a direct target of autoreactive CD4+ T cells,^24^ explaining this association.

One islet transplant study examined matching of donor and recipient at HLA-DR3 and HLA-DR4 together—resulting in 24 matches^22^ and found a shorter duration of graft function. Due to an underpowered data set, combining of haplotypes and no molecular HLA-typing the individual effects of HLA-DR3/DQ2 and HLA-DR4/DQ8 could not be ascertained.^22^ In another study examining a large cohort of simultaneous pancreas kidney recipients, matching of donor and recipient at HLA-DR3 was associated with subsequent hyperglycaemia, but matching of donor and recipient at HLA-DR4 was underpowered and statistical analyses were not performed.^25^

The mechanism by which donor HLA-DQ8 protects beta cells post-transplantation is not known. The HLA genotype has a strong influence on how antigen is presented to T lymphocytes. Antigen presenting cells include macrophages and dendritic cells which express HLA class II molecules and are part of a donor islet infusion. The affinity between the T cell receptors (TCRs) and the HLA II complex may influence T-cell differentiation and “polarize” CD4+ T cells to secrete cytokines which may post-transplant, protect against autoimmune destruction of the transplanted beta cells via for example the cytolytic pathway.^26^ Concordant with this, studies in newly diagnosed T1D people with detectable C-peptide concentrations, show that administration of DR4 (DRB1∗04:01)-restricted immunodominant proinsulin peptide preserves C-peptide concentrations.^27^ Individuals in this study most likely have DR4 in association with DQ8 typing.^23^ Alternative mechanisms that may protect the transplanted beta cells include CD8+ T cell exhaustion^28^ secondary to immunomodulation.^29^

The presence of donor HLA-DQ2∗-A05 at first transplant was associated with diminished graft survival, but not when taking into account multiple transplants per patient. For first transplants, the effect was also present where the first donor and recipient were positive for the HLA-DQ2∗-A05 antigen, but there is insufficient evidence for us to conclude that it is the matching that is associated with the adverse outcome.

Of note after adjustment for TCDs, immunosuppressive agents and other confounders the presence of HLA-DQ2∗-A05 antigen at first transplant was still associated with inferior graft outcomes, although matching between donor and recipient at first transplant for HLA-DQ2∗A05 was not statistically significant. There were no significant relationships with donor HLA-DQ2∗-A05 antigens in subsequent transplants. Induction and immunosuppressive regimens may affect the success of beta cell replacement by their effects on T cell-mediated autoimmunity and allograft rejection. The majority of transplant recipients in this study received TCDs with or without the anti-inflammatory agent, etanercept at induction. These agents are associated with improved islet transplant outcomes^20^ and in solid organ transplant with diminished rates of recurrent T1D.^25^ Alemtuzumab is associated with a reduced incidence of de novo DSA and graft rejection^30^ and decreased autoreactive T cells.^31^

At first transplantation, alemtuzumab induction occurs within one-two hours prior to transplant. It is possible that CD4+ T cells are not fully depleted at this stage and binding of the HLA-DQ2∗-A05 may polarize CD4+ T cells to secrete pro-inflammatory cytokines with autoimmune destruction of transplanted beta cells which impacts long-term graft survival.

In the earliest era of the programme, when recipient and first donor were positive for HLA-DQ2∗A05, greater graft dysfunction was seen. Evolving induction therapies and immunosuppression may play a role in protecting the graft by inducing for example regulatory T cells^16^ but this remains to be tested. Matching at this T1D susceptibility antigen in subsequent transplants did not influence graft survival and there is no evidence that avoidance of these antigens is beneficial; It may be that T cells are depleted at these subsequent transplants explaining this phenomenon. Cis and trans combinations of DQB1 and DQA1 were not taken into consideration in this analysis therefore some high-risk HLA-DQ2∗-A05 combinations may have been underappreciated however, this would have affected a small number of individuals and would have had no impact on the observed protective impact of donor HLA-DQ8. Our results suggest that HLA-DQ8 when transplanted with HLA-DQ2∗-A05 versus HLA-DQ2∗-A05 alone at first transplant may confer some graft protection but the results were of borderline significance and more data is needed. HLA-DQ2/DQ8, another independent predictor of diabetes in risk groups,^32^ was not associated with poor graft outcomes in agreement with other studies.^21^

In unadjusted analysis, the presence of HLA-A24 was associated with superior graft survival although after adjustment for confounders, this was no longer the case. HLA-A24 molecules on islet cells present preproinsulin peptide epitopes to CD8 cytotoxic T cells which we would predict may contribute to beta cell death.^33^ The mechanisms that may promote beta cell survival need further study. There was no association between donor B39 and graft survival in our study—the point estimates were large and implied a negative effect of HLA-B39 but the result was not statistically significant and there is likely to be a power issue as this antigen was prevalent at low frequency.

Importantly we have also shown that donor and islet characteristics were not significantly different between HLA-antigen groups and therefore this does not explain the results.

The impact of HLA matching in solid organ transplantation has been long established but lessened with improved immunosuppression and many allocation algorithms changed the focus from matching to wait time and medical need. However, recent data from solid organ transplants supports the importance of HLA class II matching and risk for de novo DSA and premature graft loss due to antibody mediated rejection.^34^ Recently a case series study found graft dysfunction in islet transplant recipients associated with de novo DSA^35^ underlining its importance in allograft loss. In our evaluation of DSA there was no evidence that DSA mediated the graft dysfunction in first transplants with DQ2∗A05. This finding may be explained by the high prevalence of this combination of DQ antigens in our recipient population, which prevents either DQ2 or DQA05 from being a mismatched antigen.

The benefit of islet transplantation is clear and we do not suggest that donor HLA allele expression selection and, consequently, donor/recipient matching is done based on any antigen as it is impractical and could lengthen waiting times for islet transplantation for specific individuals which could affect them adversely.

A potential confounder in this study may be the heterogeneous immunosuppression protocols used as well as numbers of islets infused but after adjustment for a number of induction and immunosuppressive agents and islet numbers over time, the data still shows a strong association with the HLA-DQ8 antigen.

GAD autoantibodies were measured pre-transplant in 43% of recipients and positive GAD autoantibody status was not more prevalent in recipients of donor HLA-DQ2∗-A05 positive islets and likewise GAD autoantibody negative status was not more prevalent in recipients of donor HLA-DQ8 positive islets and do not appear to have confounded the results. Further studies are required to understand if autoantibody surges are seen following transplantation with specific HLA antigens.

Plasma C-peptide positivity is well recorded and tends to associate with glycaemic control but does not always reflect a clinical effect and recipients that are insulin dependent may still experience hypoglycaemia and impaired awareness of hypoglycaemia even if C-peptide positive.

The strengths of the study include the largest world cohort that has been well characterised and importantly clinical practice post-transplantation was standardised. This unique HLA-antigen data set is not contained in the Collaborative Islet Transplant Registry (CITR). Furthermore, HLA typing in CITR is not detailed with no HLA-DQ typing and only small numbers available for analysis. Our findings have the potential to change islet transplant practice. While it might be desirable to select only HLA-DQ8 donors for islet transplantation based on our findings, the prevalence of only 17.5% in the general donor population would markedly impede access to donor organs and would not be practical. It is beyond the scope of this study to provide a mechanism for the protective effect of HLA-DQ8. We believe the donor HLA-DQ8 findings support further studies to examine the mechanism by which islet graft survival is improved following islet transplantation. Such further research may then lead to the development and adoption of new immunotherapies into clinical trials of beta cell preservation and replacement to extend graft longevity potentially. It may also lead to the development of different therapeutic targets for immune suppression in human islet transplantation or translate into administering specific peptides based on HLA-DQ8 to patients undergoing islet transplantation which would need evaluation in clinical trials. Our findings may impact on the selection of human embryonic stem cell derived islets from donors for transplantation in the future or even lead to modifications of gene editing stem cell derived islets: the current focus is the removal of all HLA class I and II molecules, but potentially HLA-DQ8 could, we speculate, be modified and hyper-expressed. This research could also evolve into the development of immunotherapies based on HLA-DQ8 for co-transplantation with stem cell derived islets.

New algorithms need consideration around donor selection for the first islet transplant. The immune system may not be adequately suppressed at first transplant due to the long history of autoimmunity and memory T-cell populations that may be more resistant to depletion. Our findings suggest that alternative strategies including commencement of induction therapy some time before first islet transplantation warrants consideration and further assessment.

The implications of our studies may also stimulate new avenues of research into other HLA-DQ8 associated autoimmune diseases such as coeliac disease and rheumatoid arthritis, but more research is required in this area.

In conclusion, the impact of our findings may enable pancreases to be utilised more effectively, the waiting list time for islet transplantation to decrease and our results support inclusion of HLA status in islet transplant registries. Our results will lead to improvements in patient quality-of-life with significant economic impact in the short term and will help advance further research in this field.

## Contributors

SF contributed to data interpretation, performed statistical analyses and wrote the first draft of the manuscript; AH, DG, LH and PC contributed to HLA data retrieval, verification, and interpretation, and writing of the report. AL contributed to patient follow-up, data retrieval, data interpretation, and writing of the report. DB, BA and KD, contributed to organ procurement patient care and follow-up, data retrieval, and writing of the report. DO’G and TK contributed to islet isolation, data retrieval, and writing of the report. RP performed cox survival frailty analyses and missing imputation modelling of HLA data. PAS contributed to patient follow-up, data retrieval, data interpretation, and writing of the report. AMJS contributed to organ procurement, study design, patient care and follow-up, data retrieval, data verification, data interpretation, and writing of the report. All authors had full access to all the data in the study and accept responsibility for the decision to submit for publication.

## Data sharing statement

The data underlying the results reported in this Article are not publicly available. De-identified individual participant data, as well as a data dictionary, can be made available to researchers who provide a methodologically sound proposal. Proposals should be directed to AMJS; to gain access, data requestors will need to sign a data access agreement.

## Declaration of interests

SF collaborates with and receives grant funding from Novo Nordisk Cell Therapy Programme and has received funding from charities including Medical Research Council, Diabetes UK, Chief Scientist Office, British Heart Foundation, Medical Research Scotland, EASTBIO Foundation and the Helmsley Foundation, is a member of the JDRF UK Scientific Committee, Novo Nordisk UK Research Foundation Board of Trustees and has previously served on a Diabetes UK Research Steering Committee and on a Society for Endocrinology Research Network and has previously served as a consultant to Novo Nordisk. AH has received grant funding from Canadian Institutes of Health Research, Canadian Foundation for Innovation, Canadian Society of Transplantation and has received honoraria from the Canadian Society of Transplantation and support from International Society for Transplantation. AL has received funding from the JDRF. LH is president of American Society for Histocompatibility and Immunogenetics, serves as a board member of United Network for Organ Sharing/Organ Procurement and Transplantation Network, a member of the Transplant Diagnostics Community of Practice Executive Committee and has received consultancy fees from AdaptImmune LLC and support from Thermo Fisher Scientific to attend scientific meetings. PAS has received grants from Canadian Institutes of Health Research, JDRF and Novo Nordisk, has received consultancy fees or honoraria from Abbott, Bayer, Eli Lilly, GSK, Insulet, Novo Nordisk, Sanofi, Vertex and previously served in an independent data monitoring committee overseeing safety of stem cell-derived β-cells for type 1 diabetes (Vertex Pharmaceuticals), as a board chair for Diabetes Canada, and as a co-lead for Diabetes Action Canada's innovations in a type 1 diabetes goal group. PC was previously President Of The American Society Of Histocompatibility And Immunogenetics And Previously Chair Of The Canadian Blood Services HLA Advisory Committee. AMJS has received grants or contracts from the Juvenile Diabetes Research Foundation, Canadian Stem Cell Network, Diabetes Research Foundation in Canada, ViaCyte, and the US National Institute of Diabetes and Digestive and Kidney Diseases; serves or has served as a consultant to Protokinetix, ViaCyte, Hemostemix, Pelican Therapeutics, Diagon, Aspect Biosystems and Betalin Therapeutics inc; and is a co-inventor for a patent on TNFRSF25-mediated treatments of immune diseases and disorders (PCT/US2020/053,085) and for a Cellular Transplant Site- Device-less technology (US 14/863541, CA.286512). All other authors declare no competing interests.
