# Supplementary material for: Islet transplantation outcomes in type 1 diabetes and transplantation of HLA-DQ8/DR4: results of a single-centre retrospective cohort in Canada
Source: eClinicalMedicine. 2023 Dec 13;67:102333. doi: 10.1016/j.eclinm.2023.102333 (PMC10758748; doi:10.1016/j.eclinm.2023.102333)
Supplement: Supplementary Figures and Tables [file mmc1.pdf]

Supplementary Appendix

1 Methods

1.1 Islet product release criteria

1.2 Antibody screening

1.3 Canadian cPRA calculator

1.4 Cox proportional hazards frailty survival analysis of c-peptide positive/graft survival

2 Results

2.1 Figures

Figure S1. Cumulative total IEQs and IEQ/kg received by transplant in recipients (All)

Figure S2. Frequencies of high-risk HLA antigens in islet transplant recipients and donors

Figure S3. C-peptide survival analyses – total survival and in relation to donor HLA-DQ8 with other donor HLA antigens

2.2 Tables

Table S1. GAD autoantibody status pre-transplant according to recipient HLA-DQ2*-A05 and HLA-DQ8 antigen status

Table S2A. Infusions and islet numbers transplanted in recipients

Table S2B. Islet isolation parameters

Table S3. Time between transplants

Table S4. Induction and other agents used at transplantation

Table S5. Unadjusted Hazard Ratios of HLA antigens for time to first c-peptide negative status

Table S6. Adjusted Hazard Ratios for first transplant and time to first c-peptide negative status using a standard Cox survival analysis

Table S7. Adjusted Hazard Ratios of HLA antigens with missing data imputation for time to first c-peptide negative status using a stratified Cox frailty survival analysis

1 Methods

1.1 Islet Product Release Criteria

Islet mass >4000 IEQ/kg
Viability >70% (nuclear exclusion dye staining)
Purity >30% (dithizone staining)

1.2 Antibody screening

Antibody screening assays evolved over time in the program: FlowPRA® beads were used as previously described [23] until 2010 when this was changed to a Luminex based assay LABScreen Mixed class I and II One Lambda Thermo Fisher (2010-2014) and starting in 2014 the LIFECODES® life screen deluxe (Immucor Norcross GA). A positive or borderline screen was reflexed to single antigen bead testing. LABScreen™ Single Antigen (One Lambda A Thermo Fisher Scientific Brand). All recipients had single antigen bead testing performed prior to transplant. A threshold of 1000MFI was used as the starting point for analysis but antibodies below this may be called if the pattern fits with known sensitizing antigens and, or, epitopes. Likewise, stronger antibodies may not be called if the bead reactivity does not align with known sensitizing antigens and is negative on a surrogate crossmatch. A T cell AHG crossmatch was performed prior to 2005. After single antigen bead testing was introduced in 2004, a virtual crossmatch was performed and confirmed by a cell flow crossmatch as previously described [23].

1.3 Canadian cPRA calculator

This cPRA calculator is based on the Canadian Transplant Registry. It calculates the cPRA for HLA-A, -B, -DR, -DR51/52/53, and -DQ UA; it has been further expanded to include antibodies to C locus UA also. The cPRA calculator: shorturl.at/ewPQ8 was used to generate estimates of the percentage of Canada-wide and regional Alberta wide deceased organ donors with whom a transplant candidate may be incompatible.

1.4 Cox proportional hazards frailty survival analysis of c-peptide positive / graft survival

Firstly, survival times were calculated from the time of the first transplant until time of first C-peptide negative status, or until end of observation period, whichever was sooner. For participants with one or more additional transplants before first C-peptide negative status, multiple survival times were calculated, whereby time from first transplant until each subsequent transplant was calculated separately, and in addition, the time until first C-peptide negative status (or last observation point). These survival times were then all included in the same frailty model (i.e. using the total times), and a Gaussian frailty parameter was included in the models to take into account the multiple survival times per patient. Individual transplants and end of observation period were considered as censored events. For those patients who did not have any additional transplants, we only included the survival time until first C-peptide negative status (or end of observation period if they were never C-peptide negative). In each of the frailty models we considered the separate effects of each recipient HLA-antigen. If participants had one or more HLA- antigens then they were considered as "positive" for that antigen (i.e. we did not distinguish between participants with different numbers of antigens). The same analysis was performed for the donor HLA-antigens, except that participants were regarded as "positive" for that antigen if at least one of the donors had that antigen across all previous transplants up to the event. Similarly, for matched HLA- antigens, a "positive" status was assigned for that antigen if at least one of the donors provided a match with the recipient antigen for all transplants up to the event. All frailty models were stratified by the number of transplants up to C-peptide negative status (or end of observation period), and adjusted for the cumulative number of islets up to the event as a fixed effect. Adjustment was also made for the following confounders in the model: recipient age and sex, use of T cell depleting agent ± etanercept, anakinra, MTORI+CNI and MMF. Results were reported as HRs and 95% confidence intervals, representing the risk of C-peptide negative status for each HLA-antigen.

2 Results

2.1 Figures

Figure S1. Cumulative total IEQs and IEQ/kg received by transplant in recipients (All)

Figure S1A.

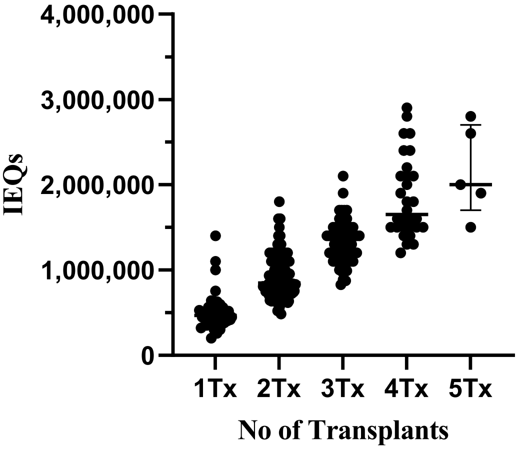

Figure S1B.

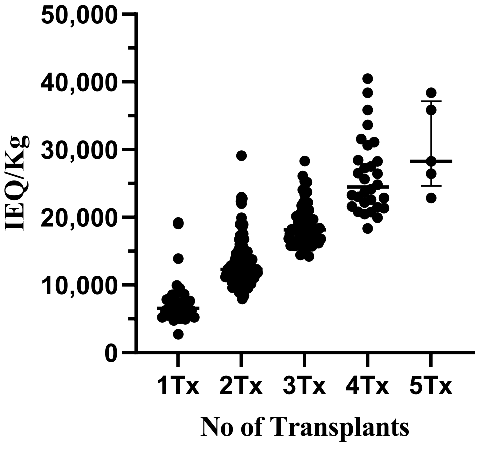

Legend: A) Cumulative total IEQs and B) IEQ/kg received in transplant recipients. Recipients received islets in 1-5 transplants. Numbers shown are those received by recipient's last transplant. Median(IQR) is indicated. Tx – transplant.

Figure S2. Frequencies of high-risk HLA antigens in islet transplant recipients and donors

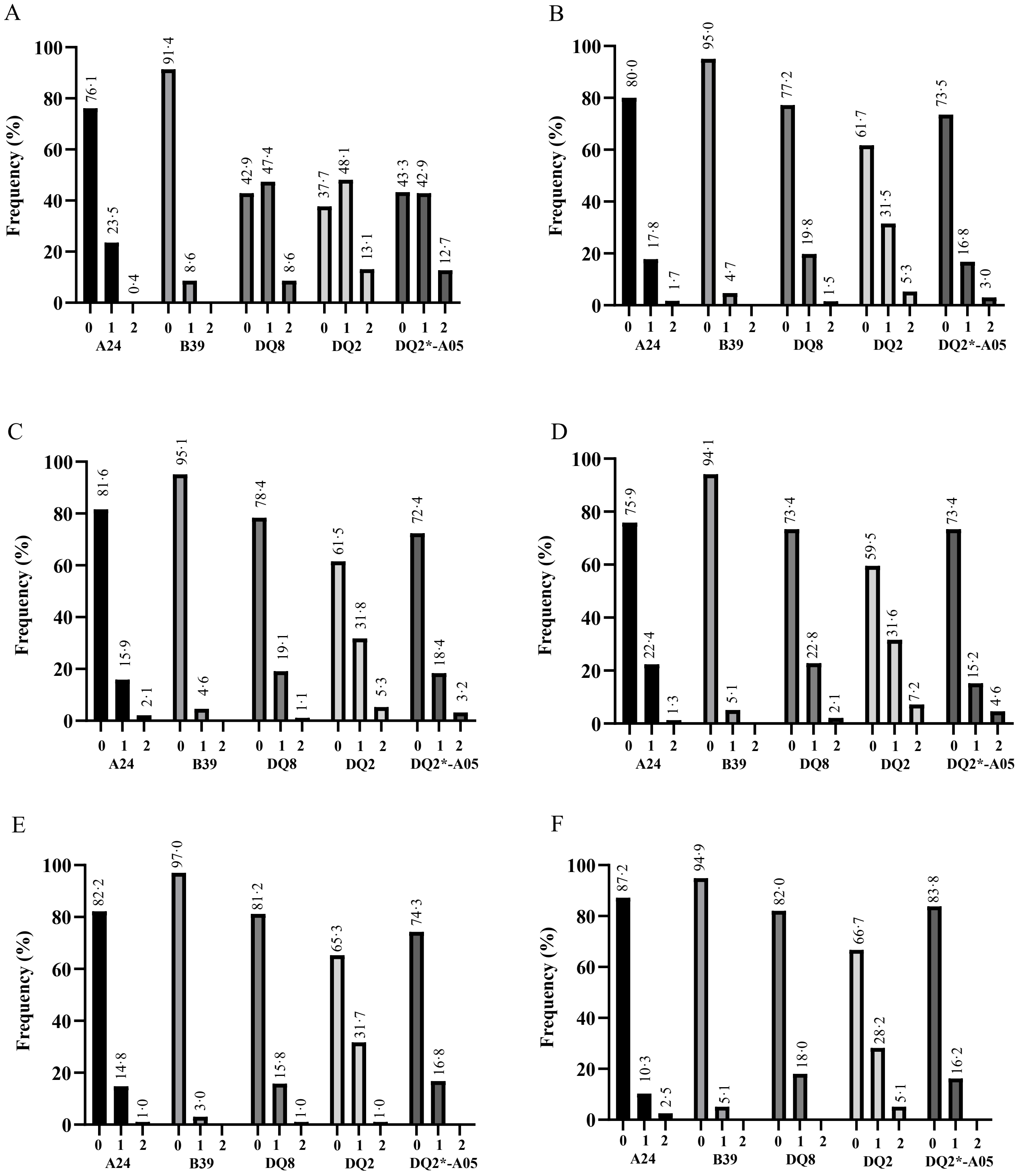

Legend: Frequencies of HLA antigens: -A24, -B39, -DQ8, -DQ2, -DQ2*-A05, in: A) Recipient HLA Antigen Frequency (ALL), B) Donor HLA Antigen Frequency (ALL), C) Donor HLA Antigen Frequency (Tx1), D) Donor HLA Antigen Frequency (Tx2), E) Donor HLA Antigen Frequency (Tx3), F) Donor HLA Antigen Frequency (Tx4-5) Transplants 4 and 5 – the data was amalgamated due to numbers. Tx – transplants. 0-1-2 are the number of HLA antigens present. Missing data: S2A (Recipient ALL) A24 (0%), B39 (0%), DQ8 (1·1%), DQ2 (1·1%), DQ2*-A05 (1·1%), S2B (Donor ALL) A24 (0·5%), B39 (0·3%), DQ8 (1·5%), DQ2 (1·5%), DQ2*-A05 (6·7%), S2C (Donor Tx1) A24 (0·3%), B39 (0·3%), DQ8 (1·4%), DQ2 (1·4%), DQ2*-A05 (6·0%), S2D (Donor Tx2) A24 (0·4%), B39 (0·8%), DQ8 (1·7%), DQ2 (1·7%), DQ2*-A05 (6·8%), S2E (Donor Tx3) A24 (2·0%), B39 (0%), DQ8 (2·0%), DQ2 (2·0%), DQ2*-A05 (8·9%), S2F (Donor Tx4-5) A24 (0%), B39 (0%), DQ8 (0%), DQ2 (0%), DQ2*-A05 (0%).
$p<0{\cdot}005$ for comparison between all HLA allele frequencies in recipients versus donors.

Figure S3. C-peptide survival analyses – total survival and in relation to donor HLA-DQ8 with other donor HLA antigens

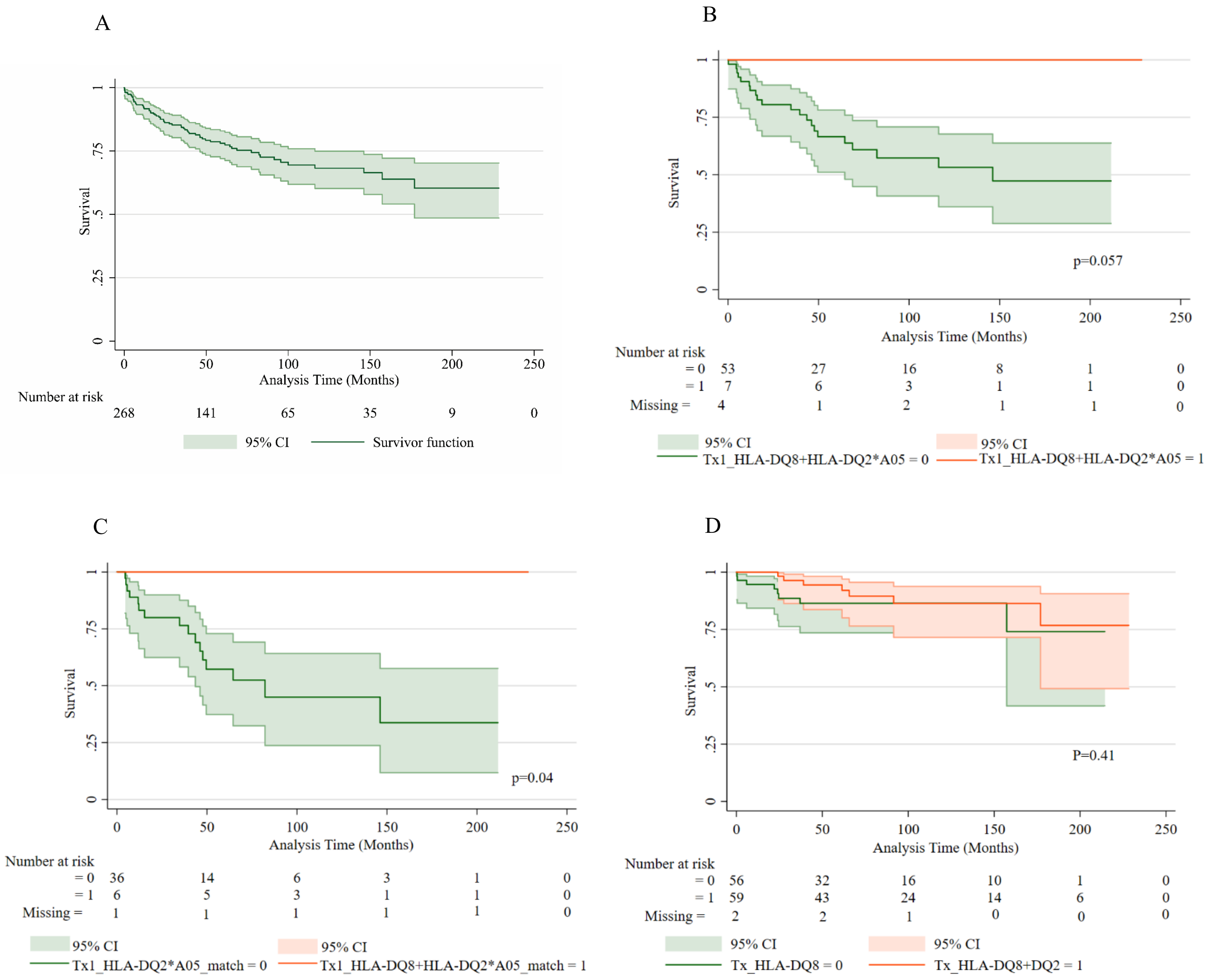

Legend: A) C-peptide Survival in Islet Transplant Recipients. Cumulative survival rates shown as proportions with 95% CI are illustrated. B-D) Kaplan Meier Survival Curves in relation to: B) 1st transplant and HLA-DQ2*A05 present in donor vs. HLA-DQ2A05 plus HLA–DQ8 in donor; green – HLA-DQ2*A05 present with no HLA-DQ8, in donor/s at first transplant, orange –HLA-DQ2*A05 present with HLA-DQ8 present in donor/s at first transplant. C) HLA-DQ2*A05 match between donor and recipient present (≥1 positive antigen in donor and recipient) across 1st transplant only vs. HLA-DQ2*A05 match between donor and recipient present at 1st transplant in addition with donor HLA-DQ8 antigen at 1st transplant – demonstrating a protective effect of -DQ8; green –match in HLA-DQ2*A05 between donor and recipient at first transplant with no donor HLA-DQ8 antigen, orange – match in HLA-DQ2*A05 between donor and recipient at first transplant with donor HLA-DQ8 antigen. D) HLA-DQ8 present (≥1 positive antigen) with no HLA-DQ2 in donors across all transplants vs HLA-DQ8 present with HLA-DQ2 in donors across all transplants; green - HLA-DQ8 present with no -DQ2 in donors across all transplants, orange - HLA-DQ8 present with HLA-DQ2 in donors across all transplants.

2.2 Tables

Table S1. GAD autoantibody status pre-transplant according to recipient HLA-DQ2*-A05 and HLA-DQ8 antigen status

**Recipient HLA**	**HLA-DQ8/ DQ2*-A05 Both –'ve**	**HLA-DQ8 +'ve**	**HLA-DQ2*-A05 +'ve**	**HLA-DQ2*-A05/DQ8 Both +'ve**
**n**	15	68	62	31
**GAD titre IU/ml**	2·9 (2·9-9·1)	10·1 (2·9-81·7)	33·2 (3·4-534·5)	51·4† (5·2-564·3)
**Proportion GAD +'ve (%)**	9·7	59·7	63·9	33·3

Legend: GAD antibody titres were available in a sample of 114 recipients of 268 in the cohort. GAD data median(IQR) is shown here in relation to recipient HLA-DQ8 and -DQ2*-A05 status. GAD positive status was taken by convention as > 5 IU/mL. In non-parametric ANOVA analyses with post-hoc testing between the groups, GAD titres were significantly greater in recipients positive for both HLA-DQ2*-A05 and HLA-DQ8 compared to: i) those recipients negative for both and ii) recipients positive for HLA-DQ8 alone; data therefore is representative of Type 1 diabetes sample. *(†, $p<0{\cdot}05$).* Positive GAD autoantibody status was not more prevalent in recipients of donor HLA-DQ2*-A05 islets and GAD autoantibody negative status was not more prevalent in recipients of donor HLA-DQ8 islets ($p>0{\cdot}05$).

Table S2A. Infusions and islet numbers transplanted in recipients

		All	ITA	IAK
**Tx1**	**N (infusions)**	268	246	22
	**N (donors)**	283	261	22
	**IEQ**	431,619 (363,762-521,823)	431,579 (363,294-519,116)	442,411 (365,333-542,931)
	**IEQ/kg**	6,080 (5,207-7055)	6,033 (5,197-6980)	6,080 (5,207-7055)
**Tx2**	**N (infusions)**	234	216	18
	**N (donors)**	239	221	18
	**IEQ**	408,523 (342,637-502,102)	408,523 (342,637-502,102)	408,338 (376,014-562,038)
	**IEQ/kg**	5,896 (5,163-6731)	5,900 (5,098-6721)	5,808 (5,368-6788)
**Tx3**	**N (infusions)**	97	93	4
	**N (donors)**	101	97	4
	**IEQ**	420,773 (369,770-484,363)	422,386 (369,770-487,907)	397,500 (371,662-427,255)
	**IEQ/kg**	5,942 (5,456-7,043)	5,963* (5,481-7,047)	5,183 (4,897-5,445)
**Tx4**	**N (infusions)**	32	31	1
	**N (donors)**	33	32	1
	**IEQ**	437,081 (366,023-531,733)	433,037 (366,023-531,733)	437,081
	**IEQ/kg**	6,082 (5,311-7,018)	6,168 (5,423-7,018)	5,311
**Tx5**	**N (infusions)**	5	5	0
	**N (donors)**	5	5	0
	**IEQ**	503,067 (462,346-695,200)	503,067 (462,346-695,200)	.
	**IEQ/kg**	7,860 (5,713-10,376)	7,860 (5,713-10,376)	.
. **Tx1-5**	**N (infusions)**	636	591	45
	**N (donors)**	661	616	45
	**IEQ**	423,933 (359,419-507,425)	424,428 (358,333-505,821)	426,849 (376,607-560,948)
	**IEQ/kg**	5,998 (5,253-6,982)	5,996 (5,237-6,983)	6,712 (5,318-7,188)

Legend: Infusions and IEQs and IEQ/kg isolated and transplanted at each transplant. Av. Tx1-5 represents the overall averages over all transplants. Numbers of islets median (IQR) transplanted in all, ITA and IAK recipients shown. Numbers received in ITA versus IAK recipients were compared. * p=0·02 for difference between ITA versus IAK. Av – average, ITA – islet transplant alone, IAK – islets after kidney, Tx – transplant.

Table S2B. Islet isolation parameters

**Islet characteristics**	
**Culture Time (hrs)**	26·0 (15-38·5)
**Islet index**	1·12 (0·91-1·32)
**Purity (%)**	60·0 (45·6-70)
**Viability (%)**	86·5 (81·5-91)

Legend: islet isolation characteristics from 661 islet isolations. Median (IQR) shown. In further analyses islet isolation characteristics were compared across all HLA-antigens and there were no significant differences according to HLA antigens (all p>0·05).

Table S3. Time between transplants

**Transplant**	**Median (IQR) (days)**	**Minimum-maximum (days)**
**1-2 ***	149(64-331)	3-3923
**2-3**	1076(297-2003)	16-4678
**3-4**	1085(222-1799)	38-6148
**4-5**	276(128-988)	119-3194

Legend: Time in days between transplants expressed as median (IQR) and minimum to maximum days.
* Interval between 1 and 2 transplants was shorter.

Table S4. Induction and other agents used at transplantation

**Transplant**	**Recipients (n)**	**Alemtuzumab**	**Etanercept**	**Alemtuzumab +/- Etanercept**	**TCD +/- Etanercept**	***Anakinra***
**1**	268	143(53·4)	158(59·0)	180(67·2)	181(67·5)	82(30·6)
**2**	234	84(35·9)	142(60·7)	151(64·5)	157(67·1)	108(46·1)
**3**	97	41(42·3)	73(75·3)	74(76·3)	74(76·3)	53(54·6)
**4**	32	17(53·1)	31(96·9)	31(96·9)	31(96·9)	24(75)
**5**	5	3(60)	5(100)	5(100)	5(100)	5(100)

Legend: Proportion of islet transplant recipients receiving induction with alemtuzumab +/- etanercept (anti-TNF alpha), other T-cell depleting agent namely thymoglobulin +/- etanercept and anakinra as part of an immunosuppression regimen. Absolute numbers (%) shown. TCD – T cell depleting agent.

Table S5. Unadjusted Hazard Ratios of HLA antigens for time to first c-peptide negative status

Subgroup	*+'ve / Total*	Hazard Ratio (95% CI)	p
**Recipient HLA antigens**			
**HLA-A24**	64/268	0·74 (0·41-1·44)	0·32
**HLA-B39**	23/268	0·84 (0·34-2·10)	0·71
**HLA-DQ8**	150/265	0·89 (0·54-1·45)	0·63
**HLA-DQ2**	164/265	0·87 (0·53-1·44)	0·61
**HLA-DQ2*A05**	149/265	1·08 (0·65-1·78)	0·77
**HLA-A24 and -B39 +'ve**	13/268	1.14 (0·42-3·15)	0·79
**HLA-DQ8, -DQ2*A05 +ve**	64/265	0·96 (0·53-1·73)	0·88
**HLA-DQ8 and -A24 +'ve**	33/268	0·86 (0·41-1·80)	0·68
**HLA-DQ8 and -B39 +'ve**	12/268	1·08 (0·34-3·46)	0·89
**HLA-DQ2*A05 and -A24 +'ve**	22/265	0·33 (0·08-1·34)	0·12
**Transplant 1 Donor HLA antigens**			
**HLA-A24**	51/268	0·35 (0·15-0·81)	**0·01**
**HLA-B39**	13/268	1.23 (0·45-3·39)	0·69
**HLA-DQ8**	56/265	0·48 (0·23-1·00)	**0·04**
**HLA-DQ2**	103/265	1·59 (0·97-2·62)	0·06
**HLA-DQ2*A05**	60/252	1·92 (1·12-3·33)	**0·02**
**Transplant 1 Recipient and Donor Matches**			
**HLA-A24 +'ve to HLA-A24 +'ve**	14/268	0·25 (0·03-1·79)	0·17
**HLA-B39 +'ve to HLA-B39 +'ve**	3/268	2·00 (0·49-8·28)	-
**HLA-DQ8 +'ve to HLA-DQ8 +'ve**	33/264	0·47 (0·17-1·28)	0·14
**HLA-DQ2 +'ve to HLA-DQ2 +'ve**	72/267	1·47 (0·88-2·46)	0·15
**HLA-DQ2*A05+'ve to HLA-DQ2*A05+'ve**	42/258	2·11 (1·19-3·75)	**0·011**
**Specified Donor Antigens (All Transplants)**			
**Donors HLA-A24 +'ve**	105/268	0·58 (0·34-0.98)	**0·04**
**Donors HLA-B39 +'ve**	29/268	1·28 (0·63-2.59)	0·49
**Donors HLA-DQ8 +'ve**	115/265	0·29 (0·16-0·53)	**<0·001**
**Donors HLA-DQ2 +'ve**	166/266	0·97 (0·58-1·63)	0·92
**Donors HLA-DQ2*A05 +'ve**	102/253	0·97 (0·56-1·65)	0·91
**Recipient to Donor Matches (All Transplants)**			
**Recipient to all Donor HLA-A24 +'ve**	32/266	0·46 (0·18-1·14)	0·10
**Recipient to all Donor HLA-B39 +'ve**	4/268	1·59 (0·39-6·54)	-
**Recipient to all Donor HLA-DQ8 +'ve**	68/263	0·34 (0·16-0·71)	**0·004**
**Recipient to all Donor HLA-DQ2 +'ve**	110/267	1·04 (0·63-1·70)	0·88
**Recipient to all Donor HLA-DQ2*A05+'ve**	67/258	1·21 (0·69-2·10)	0·51

Legend: Unadjusted HRs of HLA antigens shown for time to C-peptide negative status. The number of participants with (≥1) HLA antigen is shown across the different categories. Data inference was performed. Where there were <8 +'ve cases no statistical analyses were done. Note for transplant 1, further survival analyses is presented for HLA-DQ8 in Figure S3. Other combinations at transplant 1 also examined but limited by numbers of cases – HLA-B39 with -A24, -DQ2A05 and -DQ8 <5 cases per group.

Table S6. Adjusted Hazard Ratios for first transplant and time to first c-peptide negative status using a standard Cox survival analysis

**Donor HLA antigens (≥1 in Transplant 1)**	**+ve /Total**	**HR**	**95% CI**	**p**
**HLA**				
**HLA-DQ8 (complete cases)**	56/265	0·33	0·15 to 0·70	**0·004**
**HLA-DQ8 (MI lowest)**	57/268	0·31	0·15 to 0·67	**0·003**
**HLA-DQ8 (MI highest)**	58/268	0·42	0·21 to 0·83	**0·013**
**HLA-DQ2*A05 (complete cases)**	60/252	1·95	1·10 to 3·46	**0·022**
**HLA-DQ2*A05 (MI lowest)**	65/268	1·36	0·80 to 2·34	0·26
**HLA-DQ2*A05 (MI highest)**	71/268	2·58	1·55 to 4·31	**0·0003**
**Recipient to Donor Matched HLA antigens (≥1 in Transplant 1)**				
**HLA**				
**HLA-DQ8 (complete cases)**	33/264	0·26	0·09 to 0·75	**0·012**
**HLA-DQ8 (MI lowest)**	34/268	0·25	0·09 to 0·72	**0·010**
**HLA-DQ8 (MI highest)**	36/268	0·48	0·21 to 1·09	0·081
**HLA-DQ2*A05 (complete cases)**	42/258	1·68	0·93 to 3·02	0·083
**HLA-DQ2*A05 (MI lowest)**	45/268	1·38	0·78 to 2·45	0·27
**HLA-DQ2*A05 (MI highest)**	49/268	2·16	1·28 to 3·63	**0·004**

Legend: Adjusted Hazard ratios of HLA antigens including complete cases and Missing Imputation (MI) analyses for first transplant only with HLA-DQ8 and HLA-DQ2*A05. The model was adjusted for the confounders: islet numbers, participant age and sex, use of alemtuzumab and/or etanercept, anakinra, MTORI+CNI and MMF. The number of participants with a positive (≥1) HLA antigen or positive (≥1) recipient donor match is shown at first transplant.
Complete cases = Analysis only included valid non-missing data on antigens (i.e. no missing imputation was used). MI lowest = Analysis with the lowest possible hazard ratios assuming an extreme pattern of missing data in sensitivity analysis. MI highest = Analysis with the highest possible hazard ratios assuming an extreme pattern of missing data in sensitivity analysis. Data demonstrates protective effect of donor HLA-DQ8 on graft survival even taking into account extreme patterns of missing data and is consistent with an adverse effect of HLA-DQ2*A05 at first transplant on graft survival.

Table S7. Adjusted Hazard Ratios of HLA antigens with missing data imputation for time to first c-peptide negative status using a stratified Cox frailty survival analysis

Adjusted HRs for Recipient HLA antigens	+ve /total	HR	95% CI	p
**HLA**				
**HLA-DQ8 (MI lowest)**	152/268	0·84	0·45-1·55	0·57
**HLA-DQ8 (MI highest)**	152/268	1·01	0·55-1·86	0·97
**HLA-DQ2 (MI lowest)**	166/268	0·65	0·35-1·22	0·18
**HLA-DQ2 (MI highest)**	166/268	0·79	0·42-1·50	0·48
**HLA-DQ2*A05 (MI lowest)**	151/268	0·87	0·47-1·62	0·67
**HLA-DQ2*A05 (MI highest)**	151/268	1·06	0·57-1.98	0·85
**Adjusted HRs for Donor HLA antigens (≥1 across all donors)**				
**HLA**				
**HLA-A24 (MI lowest)**	107/268	0·65	0·33-1·26	0·20
**HLA-A24 (MI highest)**	106/268	0·75	0·39-1·46	0·40
**HLA-B39 (MI lowest)**	31/268	1·91	0·77-4·73	0·16
**HLA-B39 (MI highest)**	29/268	1·92	0·78-4·77	0·16
**HLA-DQ8 (MI lowest)**	118/268	0·30	0·15-0·60	**0·001**
**HLA-DQ8 (MI highest)**	118/268	0·42	0·22-0·81	**0·009**
**HLA-DQ2 (MI lowest)**	167/268	1·41	0·76-2·60	0·27
**HLA-DQ2 (MI highest)**	168/268	1·58	0·85-2·92	0·15
**HLA-DQ2*A05 (MI lowest)**	117/268	0·87	0·47-1·60	0·65
**HLA-DQ2*A05 (MI highest)**	112/268	2·18	1·19-3·99	**0·012**
**Adjusted HRs for Recipient and Donor Matched HLA antigens (≥1 match across all donors)**				
**HLA**				
**HLA-A24 (MI lowest)**	34/268	0·39	0·13-1·12	0·08
**HLA-A24 (MI highest)**	32/268	0·42	0·14-1·21	0·11
**HLA-DQ8* (MI lowest)**	72/268	0·28	0·12-0·68	**0·005**
**HLA-DQ8 (MI highest)**	72/268	0·55	0·26-1·18	0·12
**HLA-DQ2 (MI lowest)**	111/268	1·14	0·62-2·07	0·67
**HLA-DQ2 (MI highest)**	111/268	1·24	0·68-2·25	0·48
**HLA-DQ2*A05 (MI lowest)**	77/268	1·22	0·64-2·36	0·55
**HLA-DQ2*A05 (MI highest)**	74/268	2·39	1·29-4·42	**0·006**

Legend: Adjusted Hazard ratios of HLA antigens with Missing Imputation (MI) analyses. The model was adjusted for the confounders: islet numbers, recipient age and sex, use of T cell depleting agent ± etanercept, anakinra, MTORI+CNI. The number of participants with a positive (≥1) HLA antigen or at least 1 donor-recipient match is shown across the different categories**.** MI lowest = Analysis with the lowest possible hazard ratios assuming an extreme pattern of missing data in sensitivity analysis. MI highest = Analysis with the highest possible hazard ratios assuming an extreme pattern of missing data in sensitivity analysis. Data demonstrates protective effect of donor HLA-DQ8 on graft survival even taking into account extreme patterns of missing data.
